# Polymeric Carriers for Delivery Systems in Biomedical Applications—In Memory of Professor Andrzej Dworak

**DOI:** 10.3390/polym15081810

**Published:** 2023-04-07

**Authors:** Alicja Utrata-Wesołek, Barbara Trzebicka, Jerzy Polaczek, Iza Radecka, Marek Kowalczuk

**Affiliations:** 1 Centre of Polymer and Carbon Materials Polish Academy of Sciences, M. Curie-Sklodowskiej St. 34, 41-800 Zabrze, Poland; btrzebicka@cmpw-pan.pl; 2 SIGMA-NOT Eds., Ratuszowa St. 11, 03-450 Warszawa, Poland; 3 Faculty of Science and Engineering, University of Wolverhampton, Wolverhampton WV1 1 LY, UK; i.radecka@wlv.ac.uk

Since the appearance of the first civilizations, various substances have been used to improve human health. Although nature is undoubtedly the source of many medicinal substances, since the early 19th century there has been an enormous development of pharmaceutical products aimed at treating or improving human health. Over time, the emergence of new generations of therapeutic agents has been observed, including not only small molecules, but also proteins and peptides, monoclonal antibodies, nucleic acids, and living cells. This progress has opened up new possibilities for the use of these therapeutic agents in the treatment of specific diseases, while at the same time causing enormous challenges in delivering them into the body while maintaining their therapeutic usefulness. For the effective action of these agents, it is necessary, among other things, for them to have proper solubility, stability, target location, off-target non-toxicity, controlled pharmacokinetics, possibility of bypassing biological barriers (cell membranes, nucleus) or, in the case of cells, maintaining cell viability and phenotypes [1]. These challenges have compelled scientists to develop a variety of advanced drug delivery systems (DDSs). DDSs are formulations or devices used to transport and release (preferably in a controlled manner) therapeutic agents to their destinations in the body, and to minimize off-target drug accumulation. Thanks to such control of the rate, time, and localization of release of these drugs in the body, the DDSs enable us to achieve maximum therapeutic efficacy. The developed systems for delivering the bioactive agents include various forms, from the conventional (e.g., tablets, capsules, drops, sprays, injections, or creams) to controlled ones (smart, modulated, and targeted delivery systems in the form of micro- and nanoparticles, micelles, liposomes, polymersomes, conjugates, gels, or implants) [2,3].

Polymers, along with metals and ceramics, constitute the most common platform used for DDSs [4,5]. They have many advantages over the other classes, and many of them are well tolerated during contact with a living system without producing any adverse effects. They are non-toxic and biologically inert. Both natural and synthetic polymers can be applied for controlled drug delivery, although the use of synthetic ones is preferable. This is due to the fact that, thanks to the controlled processes of polymerization, it is possible to obtain materials with a highly reproducible structure–function relationship. Some of the polymers are biodegradable, so the problem of their accumulation within the body is eliminated, providing that their degradation by-products are non-toxic, that they do not produce any immune response, or lie below the renal threshold level. Moreover, polymers can be fabricated into complex structures and shapes (e.g., homo- and copolymers, star polymers, dendrimers, branched polymers, micelles, nanoparticles, vesicles, gels, surfaces) leading to a wide range of physicochemical properties. Last but not least, polymers have tunable chemistries, including controllable and responsive properties (e.g., stimuli responsive) and countless possibilities for modifications to achieve desired properties and mimic biological systems. All these beneficial properties of polymers have enabled huge progress in the development of drug delivery technologies, allowing for transportation of not only small drugs, both hydrophilic and hydrophobic, but also proteins and nucleic acids (Figure 1). This achievement, by improving drug safety and efficacy, may unquestionably facilitate better patient comfort and quality of life.

Over the past 70 years, DDS polymer materials and their designs have progressed from external devices and simple off-the-shelf macroscopic supplies through to microscopic particles and ultimately to complex and rationally designed nanocarriers. The application of DDSs has improved the clinical usefulness of many drugs and enabled new therapeutics, such as anti-cancer and siRNA therapies. However, there are still remaining challenges that drug delivery systems have to overcome to be clinically viable. The next generation of DDSs will need to be able to overcome the biological barriers which limit the delivery of complex therapeutic molecules, and utilize less invasive systems which secrete biomolecules into specific tissues, at specific times and concentrations, sometimes for a prolonged period of time. Therefore, the innovations in the field of polymer materials will be the driving force in overcoming existing boundaries, while the polymer DDSs will still be considered the most active field of biomedical research in pharmaceutical industries and academic laboratories.

This book comprises articles concerning recent advances in natural and synthetic polymeric materials with the desired physical, chemical, biological, and biomechanical properties to match the various requirements of controlled delivery of various therapeutic agents. The book contains reviews and original articles of eminent scientists and friends of Professor Dworak, each of which was published previously as original contributions to the *Polymers* Special Issue dedicated to the memory of late Professor Andrzej Dworak https://www.mdpi.com/journal/polymers/special_issues/polym_carr_deliv_biomed_appli_mem_prof_Dworak (accessed on 23 February 2021)

Prof. Dworak was an outstanding specialist with experience in the studies of the mechanisms of oxirane and cyclic imines polymerization and controlled radical polymerization of various types of monomers in order to obtain macromolecules with carefully planned structures and properties, including polymers sensitive to stimuli. In his research, he successfully translated knowledge in the field of polymer chemistry and polymer materials into their potential use in medicine and pharmacy. Prof. Dworak initiated many national and European projects in which he carried out studies in the field of basic research, including the preparation and therapeutic use of various types of nanoparticles and polymer nanocontainers carrying different types of active substances. He also managed projects related to the development of polymer supports for the culture and transfer of cell sheets, which significantly accelerated the healing process of burn wounds. The results of Prof. Dworak’s research has been published in nearly 150 articles in international journals, and he was the co-author of several patents.

On 17 March 2022, the Silesian Meeting on Polymer Materials Conference (Polymat 2022) was organized and dedicated to the memory of Prof. Andrzej Dworak (Figure 2). The conference was held by the Centre of Polymer and Carbon Materials of the Polish Academy of Sciences (CMPW PAN) at Zabrze on the first anniversary of Prof. Dworak’s death. The participants were welcomed by Prof. Barbara Trzebicka, Director of the Centre, and Mr. Krzysztof Lewandowski, Vice President of the City of Zabrze. After a brief opening speech delivered by the Chairman of the Committee of Chemistry of the Polish Academy of Sciences, Professor Janusz Jurczak, Professor Zbigniew Florjańczyk, Warsaw University of Technology, and Professor Stanisław Penczek, Centre of Molecular and Macromolecular Studies of Polish Academy of Sciences, Łódź, presented the work and scientific activity of Professor A. Dworak. His medicine-oriented studies on polymeric materials were emphasized in particular.

According to the conference program, Prof. Axel Müller (Figure 2), Johannes Gutenberg University Mainz, Germany, delivered a lecture on tapered block and multiblock copolymers made via statistical anionic copolymerization; Prof. Rainer Haag, Free University Berlin, Germany, lectured on the synthesis and biomedical applications of multifunctional polyglycerols; and Prof. Stergios Pispas, National Hellenic Research Foundation, Athens, Greece, presented thermoresponsive linear and hyperbranched copolymers synthesized by reversible addition-fragmentation chain-transfer (RAFT) polymerization. After a lunch break, Prof. Petar Petrov, Institute of Polymers, Bulgarian Academy of Sciences, Sofia, reviewed multifunctional block copolymer nanocarriers; and Prof. Brigitte Voit, Leibniz Institute of Polymer Research, Dresden, Germany, gave a lecture on responsive nanocapsules and multicompartments used as cellular mimics. Prof. Marek Kowalczuk (Centre of Polymer and Carbon Materials of Polish Academy of Sciences, Zabrze), Prof. Stanisław Słomkowski (Centre of Molecular and Macromolecular Studies of Polish Academy of Sciences, Łódź), Prof. Christo Tsvetanov (Institute of Polymers of Bulgarian Academy of Sciences), and Prof. Hans-Jürgen Adler (Dresden University of Technology, Germany), performed the roles of chairpersons very efficiently.

During poster sessions, more than 130 scientific communications were presented and discussed (according to the rule “one participant—one poster”). The posters were available also on the Internet. Many of them were of high practical importance. The posters were evaluated by a special jury (headed by Prof. Neli Koseva, Institute of Polymers, Bulgarian Academy of Sciences). The three best communications were selected and awarded prizes of EUR 450 each, funded by the Swiss journal *Polymers*, published by the Multidisciplinary Digital Publishing Institute. In the category of advanced synthesis and study of polymeric materials, Aneta Medaj, Jagiellonian University, Cracow, Poland, was awarded for her presentation entitled “Polymer nanocapsules templated on liquid cores as model photoreactors”. In the category of interdisciplinary and international studies, Erik Dimitrov of the Institute of Polymers, Bulgarian Academy of Sciences, Sofia, Sofia University, and the Centre of Polymer and Carbon Materials, Zabrze, Poland, was awarded for the presentation “Nucleolipid vesicles. Supramolecular structures resembling spherical nucleic acids”. In the category of advanced biopolymer materials, Mattia Parati (Figure 2), University of Wolverhampton, United Kingdom, was distinguished with an award for the paper “Algal and yeast waste fraction valorization for the biosynthesis of poly(γ-glutamic acid). A versatile biomaterial”.

The conference was held under the auspices of the European Federation of Polymers, the Committee of Chemistry of the Polish Academy of Sciences, and the Polish Chemical Society. The media patronage over the conference was taken by *Polymers*, MDPI, and the *Chemical Industry*.

The conference was accompanied with a small exhibition of research instruments (polymer analysis and testing). The Scientific Committee, headed by Prof. Zbigniew Florjańczyk, was responsible for the high scientific level of the presented papers, and the Organizing Committee, headed by Prof. Alicja Utrata-Wesołek, orchestrated the smooth running of the event. The conference was attended by about 170 scientists from Poland and abroad. After the closing remarks given by Prof. Barbara Trzebicka, chairwoman of the Conference, the participants proposed a farewell toast: to meet again at Zabrze in the near future! An extended report on the conference was also published in Polish [6].

## Figures and Tables

**Figure 1:**
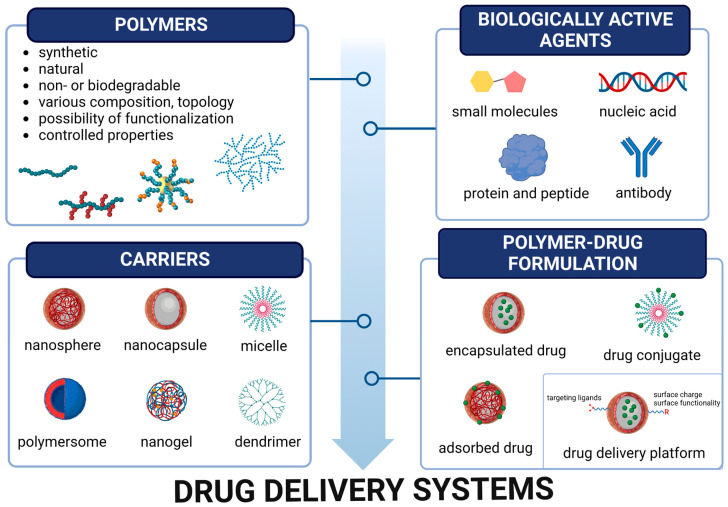
Schematic representation of the formulation of polymeric carriers for delivery systems in biomedical applications.

**Figure 2:**
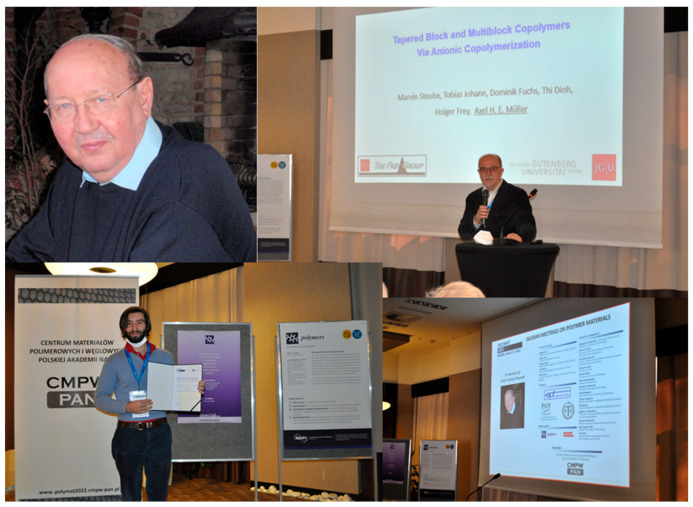
POLYMAT 2022 Conference in memory of Prof. Andrzej Dworak (photo by CMPW PAN). Top row: Prof. Andrzej Dworak; Prof. Axel Müller giving a lecture. Bottom row: Mattia Parati, University of Wolverhampton, United Kingdom, receiving the award for the best poster.
